# Complete chloroplast genome of *Mucuna birdwoodiana* (Fabaceae) and its phylogenetic analysis

**DOI:** 10.1080/23802359.2026.2746831

**Published:** 2026-10-28

**Authors:** Dan Wei, Geng Zhang, Chongyang Ran, Dafeng Li, Lei Zhang, Lingyun Yang, Huafeng Lin

**Affiliations:** ^a^ Guangdong Academy of Forestry, Guangzhou, People’s Republic of China; ^b^ Guangdong Provincial Key Laboratory of Forest Silviculture, Protection and Utilization/Key Laboratory of National Forestry and Grassland Administration on Ecosystem Conservation and Restoration in the Guangdong-Hong Kong-Macao Greater Bay Area, Guangzhou, People’s Republic of China; ^c^ Changtan Nature Reserve, Meizhou, People’s Republic of China

**Keywords:** Plastome, Fabaceae, phylogeny, IR/SC boundary, simple sequence repeat

## Abstract

*Mucuna birdwoodiana* is an ornamental and medicinal woody liana endemic to southern China. Its 153,766 bp chloroplast genome was assembled de novo from HiFi reads, recovering a typical quadripartite structure with 76 protein-coding genes, 30 tRNAs and four rRNAs. Phylogenomic analysis of 24 Fabaceae plastomes and a Polygalaceae outgroup recovered *M. birdwoodiana, M. macrocarpa*, and *M. sempervirens* as a well-supported clade. At JLA, rpl2 ends 33 or 74 bp short of the junction in *M. macrocarpa* and *M. sempervirens* but extends 1 bp into the LSC in *M. birdwoodiana*. Eighty-five simple sequence repeats (SSRs) (97.6% AT-rich) provide population-genetic markers.

## Introduction

*Mucuna birdwoodiana* Tutcher 1905 is a woody climbing legume of the genus *Mucuna*, endemic to southern China. It mainly grows in subtropical evergreen broad-leaved forests of Guangdong, Guangxi, and adjacent regions (Lu et al. 2009; Zhang et al. 2012). It is valued as an ornamental and medicinal plant, but most studies focus on its ecology and genomic resources remain limited. The lack of chloroplast genome data limits conservation, species identification, and population-genetic research on this plant. To date, four plastome sequences have been published: *M. macrocarpa* (Jin et al. 2019), *M. sempervirens* (Chen et al. 2021), *M. pruriens* (Yuan et al. 2021), and *M. manongarivensis*. These are mostly short feature reports, and no genus-wide comparative plastome analysis yet informs interspecific relationships or species delimitation in *Mucuna*.

Angiosperm chloroplast genomes are highly conserved and typically comprise a large single-copy (LSC) region, a small single-copy (SSC) region, and two inverted-repeat (IR) regions. With moderate evolutionary rates and maternal inheritance, they are suitable for species differentiation, phylogenetic analysis, and conservation genetics (Daniell et al. 2016). Here, the complete chloroplast genome of *M. birdwoodiana* was sequenced, assembled, and annotated, its phylogenetic position within Fabaceae reconstructed, and comparative analyses of IR/SC junctions and simple sequence repeats (SSRs) performed, providing genomic resources for future systematic and conservation research on *Mucuna* (Figure 1).

**Figure 1.**
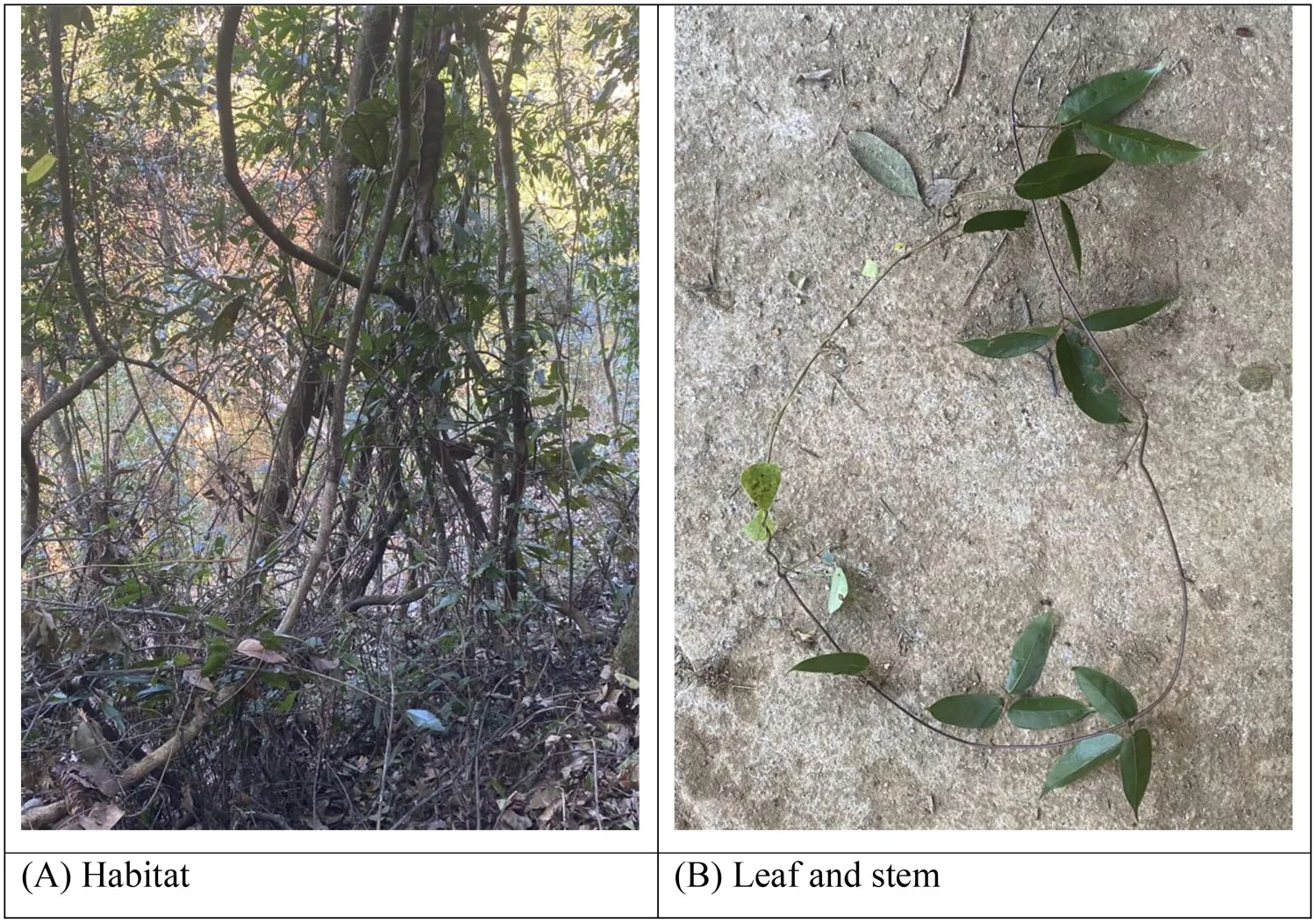
Plant morphological characteristics of *Mucuna birdwoodiana*. (A) Habitat; (B) leaf and stem. Photographed in January 2026 at Changtan Nature Reserve, Meizhou, Guangdong, China, during the non-flowering/fruiting season.

## Materials and methods

### Plant materials and sampling

Fresh leaves were collected from Changtan Nature Reserve, Meizhou, China (116.09800541°E, 24.71655823°N) on 27 January 2026. A single individual was sampled, as plastomes are highly conserved within species and one plastome suffices to establish the species-level reference. The specimen was deposited at the Guangdong Academy of Forestry (contact: Geng Zhang, gengzhang@sinogaf.cn) under the voucher number GAFMB20260127.

### DNA extraction, sequencing, assembly, and annotation

Total genomic DNA was extracted via the improved CTAB method (Doyle 1987), sequenced on the PacBio HiFi platform, and raw reads were processed with fastp v0.23.2 (Chen et al. 2018). The plastome was assembled *de novo* from total HiFi reads with OATK (Zhou et al. 2024; syncasm -k 1001 -c 30), without reference capture or post-assembly read screening, and annotated with CPGAVAS2 (Shi et al. 2019); tRNA and rRNA genes were identified via tRNAscan-SE (Lowe and Eddy 1997) and BLASTN (Chen et al. 2015), respectively. Annotations were manually applied in CPGView (Liu et al. 2023) and Apollo (Lewis et al. 2002), and the genome map was generated with OGDRAW (Greiner et al. 2019). The sequence is deposited in GenBank under accession PZ428231.1.

### Phylogenetic analysis

For phylogenetic analysis, 24 chloroplast genomes were downloaded from NCBI with *Polygala tenuifolia* as the outgroup. SSC orientation was verified with *rps*7, *ndh*F, and *ndh*H as landmarks and reverse-complemented where required (per-accession log in Supplementary File S1, Appendix A; accession quality checks in Table S3). Alignments were generated with MAFFT v7.526 (Katoh and Standley 2013). A maximum-likelihood (ML) tree was inferred in IQ-TREE v.3.1.2 (Minh et al. 2020; Wong et al. 2026), using the TVM + F + R3 model selected by ModelFinder under the Bayesian information criterion (Kalyaanamoorthy et al. 2017); branch support was assessed with 1000 SH-aLRT replicates (Guindon et al. 2010) and 10,000 UFBoot2 replicates (Hoang et al. 2018), with the -bnni option to reduce support inflation under model violation. For validation, 52 shared single-copy protein-coding genes (42,315 bp; Table S2) were codon-aligned, trimAl-trimmed (Capella-Gutiérrez et al. 2009), and concatenated in PhyloSuite v2 (Zhao et al. 2025); partitioned ML used its bundled IQ-TREE v.3.1.2 (ModelFinder BIC, edge-linked; 10,000 UFBoot/1000 SH-aLRT; Guindon et al. 2010), with two 24-taxon analyses excluding each problematic accession (Figures S4 and S5). Pairwise identities were calculated on gap-excluded sites (Table S4), and trees were visualized in iTOL v6 (Letunic and Bork 2024).

### Comparative genomic analyses

The IR/SC junction dynamics among *M. birdwoodiana*, *M. macrocarpa* (NC_044116.1), and *M. sempervirens* (NC_063832.1) were analyzed using IRscope (Amiryousefi et al. 2018). The remaining two published *Mucuna* accessions were excluded from junction comparison. *M. manongarivensis* (PZ095201.1) is only ∼53% complete, with 19 of 76 protein-coding genes missing, and *M. pruriens* (NC_054176.1) carries a truncated *ndh*K (105 bp) and a fragmented *ycf*2 annotation, precluding reliable junction comparison. The genome-wide SSRs were identified with MISA v2.1. Minimum repeat thresholds were 10/5/4/3 for mono- through hexanucleotides (Beier et al. 2017).

## Results

The circular chloroplast genome of *M. birdwoodiana* is 153,766 bp long with an overall guanine–cytosine (GC) content of 35.09% (Figure 2); its LSC, SSC, and IR regions span 87,254, 18,348, and 24,082 bp, respectively, with corresponding GC contents of 32.62%, 28.28%, and 42.16%.

**Figure 2.**
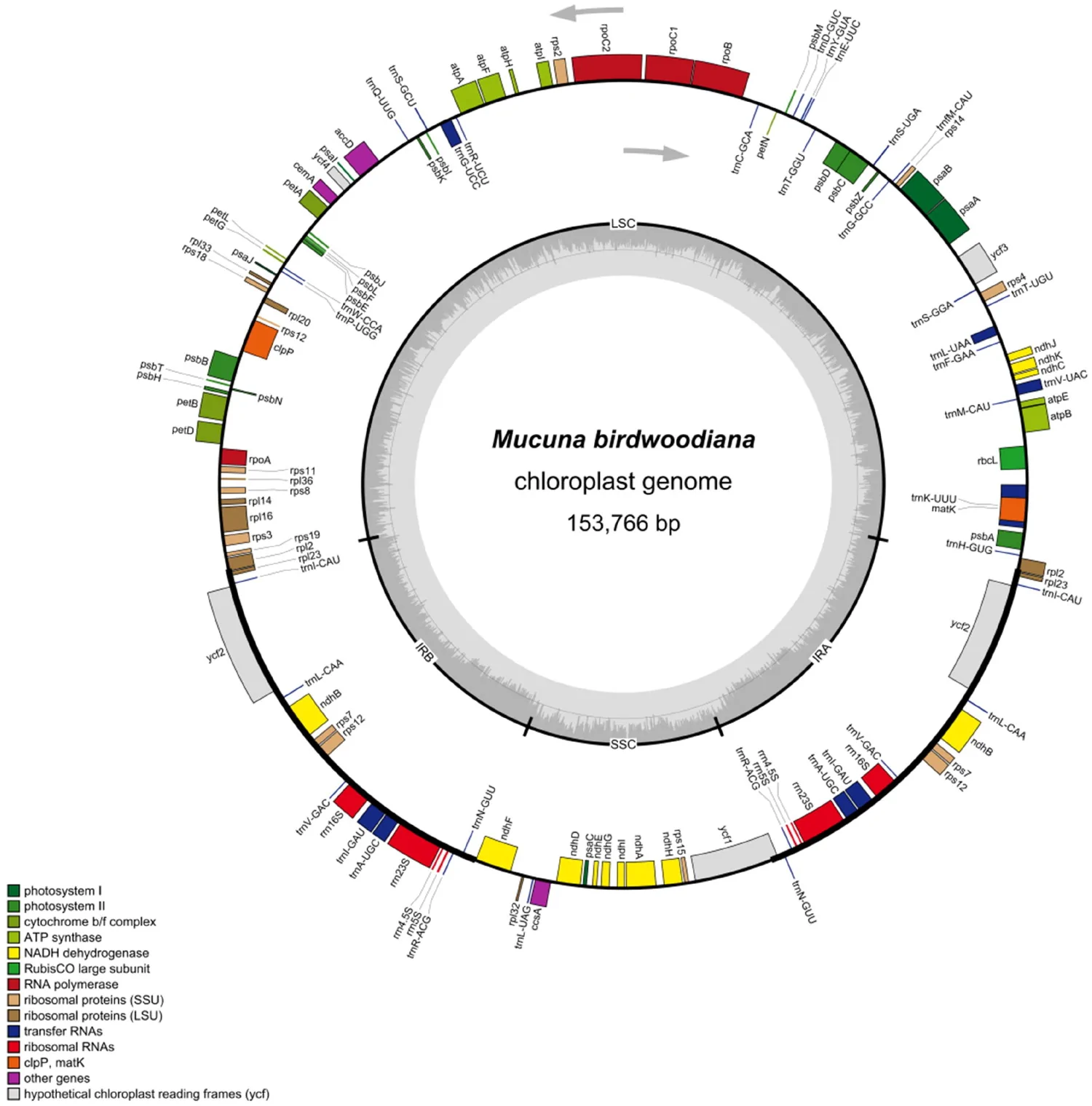
Circular chloroplast genome map of *M. birdwoodiana*. Genes outside the circle are transcribed counterclockwise; genes inside are transcribed clockwise. Dark gray inner circle indicates GC content. Functional gene groups are color-coded as labeled.

The genome contains 76 unique protein-coding genes, 30 tRNA genes, and four rRNA types (*rrn*16, *rrn*23, *rrn*4.5, *rrn*5), with six protein-coding, seven tRNA, and all four rRNA genes duplicated in the IRs (Table S1). Assembly coverage and trans-splicing gene structures are shown in Supplementary Figures S1 and S2.

The SSC-corrected 25-taxon ML tree (Figure 3) recovered a strongly supported clade of *M. birdwoodiana*, *M. macrocarpa*, and *M. sempervirens* (SH-aLRT = 100%), whereas *M. pruriens* clustered with *Cyamopsis tetragonoloba* and *Glycine* species (SH-aLRT = 93.7%) on a very short terminal branch, and *M. manongarivensis* formed a long basal branch (branch length = 0.79). In the 52-gene CDS trees, *M. pruriens* remained in the *Cyamopsis*–*Glycine* clade, but *M. manongarivensis* joined the *Mucuna* clade (SH-aLRT = 100%); excluding *M. pruriens* restored a monophyletic four-species *Mucuna* clade (SH-aLRT = 100%), whereas excluding *M. manongarivensis* did not alter the placement of *M. pruriens* (SH-aLRT = 96.1%; Supplementary Figures S3–S5; Table S5). Whole-plastome identity of *M. pruriens* was 99.7% to *Glycine max* versus 95.0% to *M. birdwoodiana*, and BLASTN searches of five full-length loci (*rbc*L, *ndh*F, *mat*K, *rpo*C1, *ycf*2) returned only *Glycine*/*Cyamopsis* accessions as top hits apart from the self-hit (Table S4).

**Figure 3.**
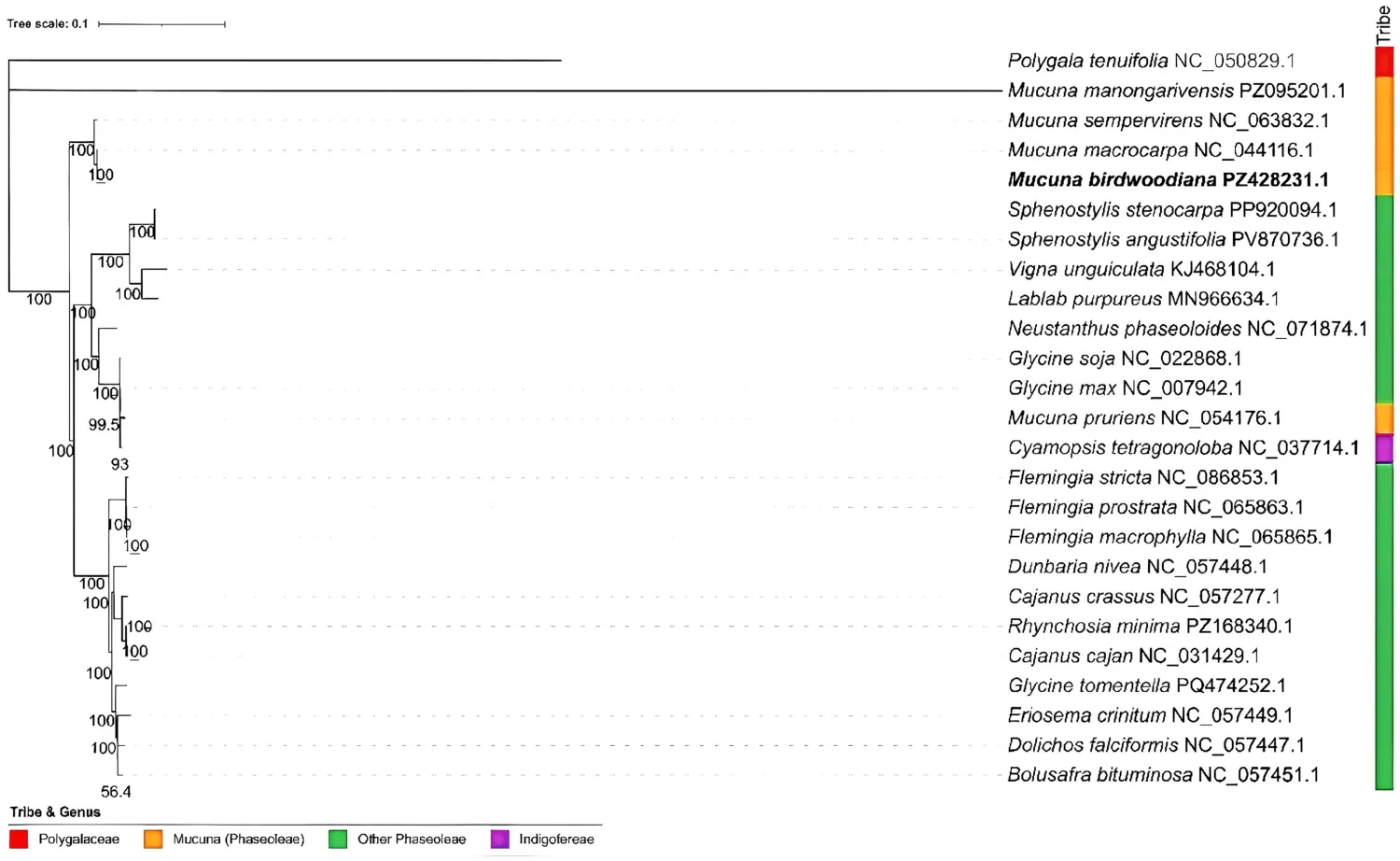
Maximum-likelihood phylogenetic tree based on 24 Fabaceae plastomes after SSC-orientation verification and correction (MAFFT v7.526; IQ-TREE v.3.1.2), rooted with *Polygala tenuifolia* (NC_050829.1; Polygalaceae) as the outgroup. The best-fit TVM + F + R3 model was selected by ModelFinder (BIC); SH-aLRT and UFBoot values (1000 and 10,000 replicates, respectively, shown as SH-aLRT/UFBoot) are given at the nodes, and the -bnni option was applied. The *Mucuna birdwoodiana* (PZ428231.1) sequence obtained in this study is shown in bold. The scale bar represents 0.1 nucleotide substitutions per site. *M. pruriens* (NC_054176.1) clusters with *Cyamopsis*/*Glycine* and shares 99.7% whole-plastome identity with *Glycine max*, whereas *M. manongarivensis* (PZ095201.1) forms a long basal branch; the two accessions are examined in Supplementary Figures S3–S5. The following sequences were used: *Polygala tenuifolia* (NC_050829.1) (outgroup), *Mucuna manongarivensis* (PZ095201.1) (unpublished), *Mucuna sempervirens* (NC_063832.1) (Chen et al. 2021), *Mucuna macrocarpa* (NC_044116.1) (Jin et al. 2019), *Mucuna birdwoodiana* (PZ428231.1) (this study), *Sphenostylis stenocarpa* (PP920094.1) (unpublished), *Sphenostylis angustifolia* (PV870736.1) (Zhang et al. 2026), *Vigna unguiculata* (KJ468104.1) (unpublished), *Lablab purpureus* (MN966634.1) (Oyebanji et al. 2020), *Neustanthus phaseoloides* (NC_071874.1) (unpublished), *Glycine soja* (NC_022868.1) (Gao and Gao 2017), *Glycine max* (NC_007942.1) (Saski et al. 2005), *Mucuna pruriens* (NC_054176.1) (Yuan et al. 2021), *Cyamopsis tetragonoloba* (NC_037714.1) (Kaila et al. 2017), *Flemingia stricta* (NC_086853.1) (Cai et al. 2024), *Flemingia prostrata* (NC_065863.1) (Wei et al. 2025), *Flemingia macrophylla* (NC_065865.1) (Wei et al. 2025), *Dunbaria nivea* (NC_057448.1) (Oyebanji et al. 2020), *Cajanus crassus* (NC_057277.1) (Oyebanji et al. 2020), *Rhynchosia minima* (PZ168340.1) (unpublished), *Cajanus cajan* (NC_031429.1) (unpublished), *Glycine tomentella* (PQ474252.1) (unpublished), *Eriosema crinitum* (NC_057449.1) (Oyebanji et al. 2020), *Dolichos falciformis* (NC_057447.1) (Oyebanji et al. 2020), and *Bolusafra bituminosa* (NC_057451.1) (Oyebanji et al. 2020).

**Figure 4.**
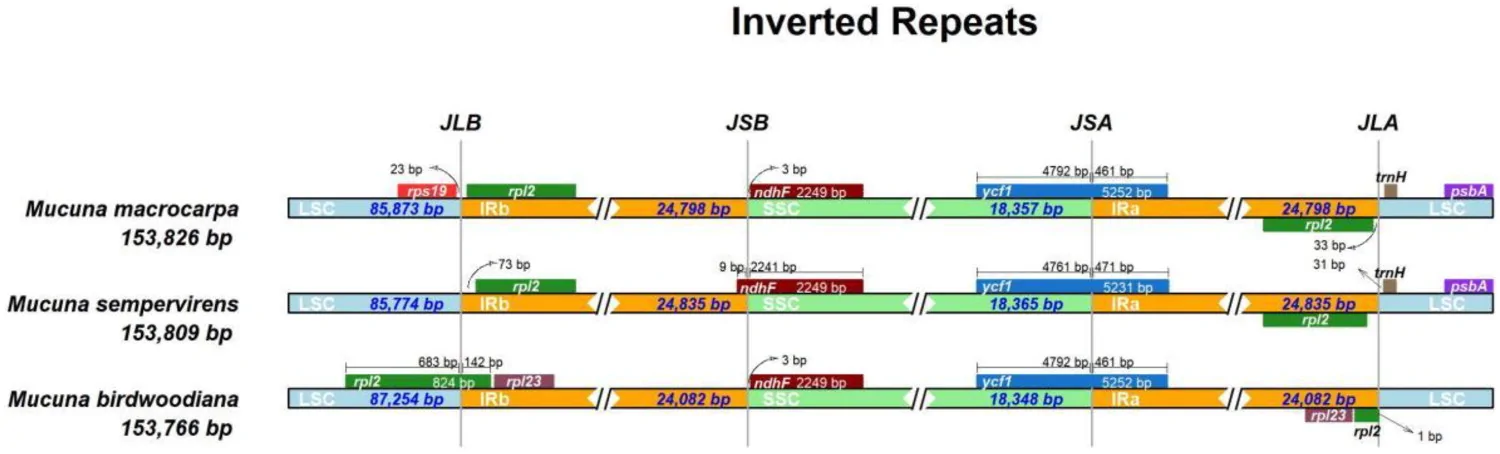
Comparison of IR/SC boundary dynamics among three *Mucuna* species. The four junctions (JLB, JSB, JSA, and JLA) show the boundaries between LSC and IRb, SSC and IRb, SSC and IRa, and LSC and IRa, respectively. Genes transcribed in the forward orientation are placed below the genome line, whereas those transcribed in the reverse orientation are shown above it.

JSB and JSA boundaries were highly conserved, whereas the JLA junction showed pronounced divergence: *rpl2* terminated 33 and 74 bp short of JLA within the IRa in *M. macrocarpa* and *M. sempervirens*, respectively, but extended 1 bp across JLA into the LSC in *M. birdwoodiana*, identifying JLA as an IR contraction/expansion hotspot.

Eighty-five SSRs were detected (Table 1), dominated by mononucleotide repeats, with a strong A/T bias, providing population-genetic markers.

**Table 1.** Summary of SSRs identified in the chloroplast genome of *Mucuna birdwoodiana.*

SSR type	Number of SSRs	Proportion (%)	Motif(s) (count)	Repeat length range (bp)
Mononucleotide	54	63.5	A (27), T (25), C (1), G (1)	10–16
Dinucleotide	29	34.1	AT (16), TA (13)	12–18
Trinucleotide	2	2.4	TTA (2)	15
Total	85	100	–	–
Compound SSRs	8	–	–	–

## Discussion and conclusions

The *M. birdwoodiana* plastome shows the typical quadripartite structure and a gene content highly similar to *M. macrocarpa* (Jin et al. 2019) and *M. sempervirens* (Chen et al. 2021), indicating conserved plastome evolution within *Mucuna*.

Phylogenetic analyses place *M. birdwoodiana* in a well-supported clade with *M. macrocarpa* and *M. sempervirens*, and *Mucuna* is recovered as monophyletic (SH-aLRT = 100%) once the problematic *M. pruriens* (NC_054176.1) is excluded; its apparent non-monophyly in the 25-taxon tree is therefore a data-quality artifact rather than a true relationship. *M. pruriens* (NC_054176.1) shares 99.7% of its plastome with *Glycine max*, and eight genes (*rbc*L, *ndh*F, *rpo*B, *psb*A, *atp*A, *ycf*2, *rpo*C1, *mat*K) are 97–100% identical to soybean (five loci by BLASTN; three by pairwise alignment) but only 90–98% similar to *Mucuna*. Its truncated *ndh*K (105 bp) and fragmented *ycf*2 indicate contamination, reference-guided assembly bias, or misidentification resolvable only by re-sequencing. The incomplete *M. manongarivensis* causes long-branch attraction only in whole-plastome analyses; shared-gene analyses restore it to the *Mucuna* clade (∼98% shared-gene identity, SH-aLRT = 100% support) (Table S5). These findings underscore the need to verify GenBank accessions and broaden *Mucuna* sampling.

The JLA junction represents the primary hotspot of IR/SC contraction/expansion in *Mucuna*, and comparable lineage-specific shifts occur in other phaseoloid legumes (Yen et al. 2023). The 85 AT-biased SSRs provide population-genetic markers (Nguyen et al. 2025) and support future systematic and conservation research.

## Supplementary Material

Supplemental Material

## Data Availability

The genome sequence data that support the findings of this study are openly available in GenBank of NCBI at https://www.ncbi.nlm.nih.gov under the accession no. PZ428231.1. The associated BioProject, Sequence Read Archive (SRA), and BioSample numbers are PRJNA1503751, SRR39909428, and SAMN62030017, respectively.
