# Supplementary material for: Complete chloroplast genome of Mucuna birdwoodiana (Fabaceae) and its phylogenetic analysis
Source: Mitochondrial DNA B Resour. 2026 Oct 28;11(11):1364–9. doi: 10.1080/23802359.2026.2746831 (PMC13721816; doi:10.1080/23802359.2026.2746831)
Supplement: Supplementary file 1 [file TMDN_A_2746831_SM8120.docx]

**Supplementary Materials**

**Figure S1.** Sequencing depth and coverage map of the chloroplast genome. The two IR regions show approximately twice the coverage of the SSC region due to reads stacking from identical copies during assembly. The depth peak observed within the LSC region is attributed to local sequence characteristics (e.g., repetitive sequences or rRNA gene clusters) and does not affect the overall assembly quality. The minimum coverage across the genome is 42.0×, well above the threshold for reliable assembly.

**Figure S2.** Schematic representation of cis-splicing (A) and trans-splicing (B) genes in the chloroplast genome of *Mucuna birdwoodiana*. Exons are shown in black, introns in white, and arrows indicate the direction of gene transcription. The diagram was generated using CPGView (<http://www.1kmpg.cn/cpgview>).

**Figure S3.** Maximum-likelihood tree of 25 taxa from 52 concatenated chloroplast protein-coding genes (42,315 bp; 133 partitions; IQ-TREE via PhyloSuite v2), with SH-aLRT support values (%) shown at the nodes.

**Figure S4.** Maximum-likelihood tree of 24 taxa after excluding NC_054176.1, showing a monophyletic four-species *Mucuna* clade (SH-aLRT = 100%).

**Figure S5.** Maximum-likelihood tree of 24 taxa after excluding PZ095201.1 (*M. manongarivensis*); *M. pruriens* remains clustered with *Cyamopsis/Glycine* (SH-aLRT = 96.1), showing that its placement is independent of the incomplete *M. manongarivensis* record.

**Supplementary Tables S1–S5**

**Table S1. Gene composition of the *Mucuna birdwoodiana* chloroplast genome (PZ428231.1)**

| **Category** | **Gene group** | **Gene name** | **No.** |
| --- | --- | --- | --- |
| Photosynthesis | Photosystem I | *psa*A, *psa*B, *psa*C, *psa*I, *psa*J | 5 |
| Photosynthesis | Photosystem II | *psb*A, *psb*B, *psb*C, *psb*D, *psb*E, *psb*F, *psb*H, *psb*I, *psb*J, *psb*K, *psb*L, *psb*M, *psb*N, *psb*T, *psb*Z | 15 |
| Photosynthesis | Cytochrome b6/f complex | *pet*A, *pet*B*, *pet*D*, *pet*G, *pet*L, *pet*N | 6 |
| Photosynthesis | ATP synthase | *atp*A, *atp*B, *atp*E, *atp*F*, *atp*H, *atp*I | 6 |
| Photosynthesis | NADH dehydrogenase | *ndh*A*, *ndh*B*(2), *ndh*C, *ndh*D, *ndh*E, *ndh*F, *ndh*G, *ndh*H, *ndh*I, *ndh*J, *ndh*K | 11 |
| Photosynthesis | R*ubis*CO large subunit | *rbc*L | 1 |
| Self-replication | Ribosomal proteins, LSU | *rpl*14, *rpl*16*, *rpl*2*(2), *rpl*20, *rpl*23(2), *rpl*32, *rpl*33, *rpl*36 | 8 |
| Self-replication | Ribosomal proteins, SSU | *rps*11, *rps*12†(2), *rps*14, *rps*15, *rps*18, *rps*19, *rps*2, *rps*3, *rps*4, *rps*7(2), *rps*8 | 11 |
| Self-replication | RNA polymerase | *rpo*A, *rpo*B, *rpo*C1*, *rpo*C2 | 4 |
| Other genes | Acetyl-CoA carboxylase / c-type cytochrome / envelope membrane | *acc*D, *ccs*A, *cem*A | 3 |
| Other genes | ATP-dependent protease / maturase | *clp*P**, *mat*K | 2 |
| Unknown function | Conserved open reading frames | *ycf*1, *ycf*2(2), *ycf*3**, *ycf*4 | 4 |
| Ribosomal RNA | rRNA genes (all duplicated in IRb/IRa) | *rrn*4.5S(2), *rrn*5S(2), *rrn*16S(2), *rrn*23S(2) | 4 (8) |
| Transfer RNA | tRNA genes | *trn*A-UGC(2), *trn*C-GCA, *trn*D-GUC, *trn*E-UUC, *trn*F-GAA, *trn*G-GCC, *trn*G-UCC, *trn*H-GUG, *trn*I-CAU(2), *trn*I-GAU(2), *trn*K-UUU, *trn*L-CAA(2), *trn*L-UAA, *trn*L-UAG, *trn*M-CAU, *trn*N-GUU(2), *trn*P-UGG, *trn*Q-UUG, *trn*R-ACG(2), *trn*R-UCU, *trn*S-GCU, *trn*S-GGA, *trn*S-UGA, *trn*T-GGU, *trn*T-UGU, *trn*V-GAC(2), *trn*V-UAC, *trn*W-CCA, *trn*Y-GUA, *trnf*M-CAU | 30 (37) |

Totals: 76 unique protein-coding genes (82 CDS copies; *ndh*B, *rpl*2, *rpl*23, *rps*7, *rps*12 and *ycf*2 duplicated in the IRs), 30 distinct tRNAs (37 copies; seven duplicated in the IRs), and four rRNA types (eight copies). Region boundaries (circular annotation): LSC 1–87,254 bp; IRb 87,255–111,336 bp; SSC 111,337–129,684 bp; IRa 129,685–153,766 bp.

**Table S2. The 52 shared single-copy protein-coding genes used in the CDS analyses and the genes excluded from the zero-missingness matrix.**

**A. Retained genes (52; 42,315 bp concatenated): shared by all 25 taxa, single-copy, and without internal stops; extracted and codon-aligned in P*hylo*S*uite* v2 (MAFFT; trimAl gappyout). Per-gene aligned lengths are given in the accompanying spreadsheet.**

| *atp*A | *atp*B | *atp*E | *atp*H |
| --- | --- | --- | --- |
| *atp*I | *ccs*A | *ndh*C | *ndh*D |
| *ndh*E | *ndh*F | *ndh*G | *ndh*H |
| *ndh*I | *ndh*J | *ndh*K | *pet*A |
| *pet*B | *pet*D | *psa*A | *psa*B |
| *psa*C | *psa*I | *psb*A | *psb*B |
| *psb*C | *psb*D | *psb*E | *psb*F |
| *psb*H | *psb*I | *psb*K | *psb*T |
| *psb*Z | *rbc*L | *rpl*14 | *rpl*16 |
| *rpl*2 | *rpl*20 | *rpl*23 | *rpl*36 |
| *rpo*A | *rpo*B | *rpo*C1 | *rps*11 |
| *rps*14 | *rps*18 | *rps*2 | *rps*3 |
| *rps*4 | *rps*7 | *rps*8 | *ycf*2 |

**B. Excluded genes, grouped by reason.**

| **Reason for exclusion** | **Genes / entries** |
| --- | --- |
| Absent only in the incomplete *M. manongarivensis* record (recoverable by re-annotation) | *atp*F, *cem*A, *mat*K, *ndh*B, *pet*G, *pet*L, *psa*J, *psb*M, *psb*N, *rpo*C2, *rps*15, *ycf*3 |
| Absent in *M. manongarivensis* and one other taxon (IR boundary or lineage loss) | *acc*D (also outgroup *Polygala*), *rpl*32 (also *C. cajan*), *rps*12 (also *C. cajan*; trans-spliced), *rps*19 (also *M. sempervirens*), *ycf*1 (also *S. stenocarpa*; pseudogene-prone) |
| Taxon-specific annotation gaps in other accessions | *clp*P (*Polygala*), *ndh*A (*M. sempervirens*), *pet*N (*C. cajan*), *psb*J (*F. stricta*), *psb*L (*S. stenocarpa*, *M. sempervirens*) |
| Insufficient occupancy across taxa | *rpl*33 and *ycf*4 (19/25), *rps*16 (16/25), *inf*A (1/25; nuclear transfer), *ycf*15 (1/25; pseudogene), *psb*G (synonym of *ndh*K; duplicate) |
| Duplicate IR copies and unnamed ORFs (not orthologous across taxa) | second IR copies of *ndh*B, *rpl*2, *rpl*23, *rps*7, *rps*12, *ycf*2, *ycf*1, *rps*19 and *ndh*A/*ndh*H/*ndh*I/*rps*15/*ycf*15; *orf*188, *orf*42, *orf*56, *paf*I |

**C. Partitioning and model selection (M*odel*F*inder*, BIC, edge-linked, IQ-TREE via P*hylo*S*uite* v2).**

| **Item** | **Value** |
| --- | --- |
| Genes in concatenated matrix | 52 |
| Codon-position subsets before merging | 52 × 3 = 156 |
| Final merged partitions (charsets) | 133 |
| Distinct substitution models | 38; TPM3u+F most frequent (18 partitions) |
| Branch lengths | edge-linked across partitions |
| Support assessment | SH-aLRT (1,000) + UFBoot2 (10,000) |

**Table S3. The 25 plastome accessions used: length, gene counts, completeness, SSC-orientation treatment and quality-control notes**

| **No.** | **Species** | **Accession** | **Role** | **Length (bp)** | **C/T/R** | **Complete (%)** | **SSC treatment** | **QC note** |
| --- | --- | --- | --- | --- | --- | --- | --- | --- |
| 1 | *Mucuna birdwoodiana* | PZ428231.1 | Ingroup, *Mucuna* | 153,766 | 76/30/4 | 100 | Forward; unchanged | Focal species; P*ac*B*io* HiFi de novo assembly |
| 2 | *Mucuna macrocarpa* | NC_044116.1 | Ingroup, *Mucuna* | 153,826 | 76/30/4 | 100 | Forward; unchanged | Pass; full circular record |
| 3 | *Mucuna sempervirens* | NC_063832.1 | Ingroup, *Mucuna* | 153,809 | 73/29/4 | 100 | Forward; unchanged | Pass; *ndh*A, *psb*L, *rps*19 not annotated |
| 4 | *Mucuna manongarivensis* | PZ095201.1 | Ingroup, *Mucuna* | 81,837 | 59/11/4 | 53.2 | Not assessed (single *rps*7); as-is | Incomplete: 19/76 PCGs, 19 tRNAs absent; single *rps*7; excluded from junction test; long branch (Fig. 3) |
| 5 | *Mucuna pruriens* | NC_054176.1 | Ingroup, *Mucuna* | 152,119 | 81/21/4 | 100 | Reverse; reverse-complemented | Soybean-like (99.68% to *G. max*; Table S4); *ndh*K 105 bp, *ycf*2 fragmented; excluded from junction test |
| 6 | *Cajanus cajan* | NC_031429.1 | Ingroup | 153,841 | 74/28/4 | 100 | Reverse; reverse-complemented | Pass; *pet*N, *rpl*32, *rps*12 not annotated |
| 7 | *Cajanus crassus* | NC_057277.1 | Ingroup | 153,549 | 77/30/4 | 100 | Forward; unchanged | Pass; full circular record |
| 8 | *Cyamopsis tetragonoloba* | NC_037714.1 | Ingroup | 152,530 | 79/30/4 | 100 | Reverse; reverse-complemented | Pass; *ycf*4 absent |
| 9 | *Dolichos falciformis* | NC_057447.1 | Ingroup | 153,884 | 77/30/4 | 100 | Forward; unchanged | Pass; full circular record |
| 10 | *Dunbaria nivea* | NC_057448.1 | Ingroup | 153,121 | 77/30/4 | 100 | Forward; unchanged | Pass; full circular record |
| 11 | *Eriosema crinitum* | NC_057449.1 | Ingroup | 153,403 | 77/30/4 | 100 | Forward; unchanged | Pass; full circular record |
| 12 | *Bolusafra bituminosa* | NC_057451.1 | Ingroup | 153,540 | 77/30/4 | 100 | Forward; unchanged | Pass; full circular record |
| 13 | *Flemingia macrophylla* | NC_065865.1 | Ingroup | 152,937 | 76/30/4 | 100 | Forward; unchanged | Pass; *rpl*33 not annotated |
| 14 | *Flemingia prostrata* | NC_065863.1 | Ingroup | 153,033 | 76/30/4 | 100 | Forward; unchanged | Pass; *rpl*33 not annotated |
| 15 | *Flemingia stricta* | NC_086853.1 | Ingroup | 152,940 | 75/30/4 | 100 | Forward; unchanged | Pass; *psb*J, *rpl*33 not annotated |
| 16 | *Glycine max* | NC_007942.1 | Ingroup | 152,218 | 76/31/4 | 100 | Reverse; reverse-complemented | Pass; *ycf*4 absent |
| 17 | *Glycine soja* | NC_022868.1 | Ingroup | 152,217 | 76/30/4 | 100 | Reverse; reverse-complemented | Pass; *ycf*4 absent |
| 18 | *Glycine tomentella* | PQ474252.1 | Ingroup | 153,855 | 76/30/4 | 100 | Reverse; reverse-complemented | Pass; *ycf*4 absent |
| 19 | *Lablab purpureus* | MN966634.1 | Ingroup | 151,709 | 76/30/4 | 100 | Forward; unchanged | Pass; full circular record |
| 20 | *Neustanthus phaseoloides* | NC_071874.1 | Ingroup | 153,192 | 78/28/4 | 100 | Forward; unchanged | Pass; full circular record |
| 21 | *Rhynchosia minima* | PZ168340.1 | Ingroup | 153,846 | 77/30/4 | 100 | Forward; unchanged | Pass; *clp*P absent |
| 22 | *Sphenostylis angustifolia* | PV870736.1 | Ingroup | 149,636 | 75/30/4 | 100 | Reverse; reverse-complemented | Pass; *rpl*33 not annotated |
| 23 | *Sphenostylis stenocarpa* | PP920094.1 | Ingroup | 149,622 | 73/29/4 | 100 | Reverse; reverse-complemented | Pass; nine CDS (incl. atp cluster) not annotated |
| 24 | *Vigna unguiculata* | KJ468104.1 | Ingroup | 151,866 | 76/29/4 | 100 | Forward; unchanged | Pass; *rpl*33 not annotated |
| 25 | *Polygala tenuifolia* | NC_050829.1 | Outgroup (*Polygala*ceae) | 165,423 | 74/29/4 | 100 | Conflicting *ndh*F/*ndh*H strands; not corrected | Outgroup; *acc*D and *clp*P absent; SSC not corrected |

**Table S4. Sequence-identity and BLASTN evidence for the anomalous *Mucuna pruriens* NC_054176.1 record.**

**A. Key pairwise identities (gap-excluded). WG, whole-plastome 210,251-bp alignment; CDS, 42,315-bp concatenation of 52 shared genes. Full 25×25 matrices are in the accompanying spreadsheet.**

| **Comparison** | **Identity (%)** |
| --- | --- |
| *M. pruriens* – *Glycine max* (NC_007942.1), WG | 99.68 |
| *M. pruriens* – *Glycine soja* (NC_022868.1), WG | 99.66 |
| *M. pruriens* – *Cyamopsis tetragonoloba* (NC_037714.1), WG | 99.60 |
| *M. pruriens* – *M. birdwoodiana* (PZ428231.1), WG | 95.04 |
| *M. birdwoodiana* – *M. macrocarpa*, WG | 99.92 |
| *M. birdwoodiana* – *M. sempervirens*, WG | 99.51 |
| *M. birdwoodiana* – *M. manongarivensis*, WG (68,661 comparable sites only) | 67.87 |
| *M. manongarivensis* – *M. birdwoodiana*, CDS | 98.04 |
| *M. manongarivensis* – *M. macrocarpa* / *M. sempervirens*, CDS | 98.07 / 98.07 |
| *M. birdwoodiana* – *M. macrocarpa*, CDS | 99.96 |

**B. Eight protein-coding loci of *M. pruriens*: pairwise identity to *Glycine max* and *M. birdwoodiana* (Needleman–Wunsch global alignment, ungapped columns; *mat*K reverse-complemented; *rpo*C1 includes its intron; *ycf*2 uses the 6,864-bp main fragment).**

| **Locus** | **Length (bp)** | **vs *G. max* (%)** | **vs *M. birdwoodiana* (%)** | **Independent BLASTN (Table S4-C)** |
| --- | --- | --- | --- | --- |
| *rbc*L | 1,428 | 100.00 | 96.78 | 100% cover / 100% identity, E=0 |
| *ndh*F | 2,241 | 100.00 | 94.11 | 100% cover / 100% identity, E=0 |
| *rpo*B | 3,213 | 100.00 | 96.58 | Pairwise only (not BLAST-tested) |
| *psb*A | 1,062 | 100.00 | 98.21 | Pairwise only (not BLAST-tested) |
| *atp*A | 1,533 | 100.00 | 97.20 | Pairwise only (not BLAST-tested) |
| *ycf*2 (main fragment) | 6,864 | 100.00 | 97.92 | 100% cover / 100% identity, E=0; score 26,415 (> self-hit 25,396) |
| *rpo*C1 | 2,862 | 97.96 | 95.56 | 100% cover / 98.04% identity, E=0 |
| *mat*K | 1,512 | 97.22 | 89.95 | 100% cover / 97.35% identity, E=0 |

*Five full-length loci are BLASTN-confirmed at 97.35–100% (rpo*B*, psb*A *and atp*A *were assessed by pairwise alignment only).*

**Table S4-C. NCBI BLASTN verification of selected loci (run 18 September 2026)**

| **Query (accession, locus, length)** | **RID** | **Top non-self hits (accession)** | **Cover** | **Identity** | **E-value** | **Interpretation** |
| --- | --- | --- | --- | --- | --- | --- |
| *M. pruriens* NC_054176.1, *rbc*L, 1,428 bp | AT90XM1P014 | *G. soja* (PV685018.1, PZ683366.1), *G. max* (MW357265.1); *C. tetragonoloba* NC_037714.1 | 100% | 100.00% | 0 | Identical to soybean *rbc*L; no *Mucuna among* top hits |
| *M. pruriens* NC_054176.1, *ndh*F, 2,241 bp | AT90XM1P014 | *G. soja* PZ683366.1, *G. max* MZ964143.1, *C. tetragonoloba* NC_037714.1, *G. gracilis* NC_030329.1 | 100% | 100.00% | 0 | Identical to soybean *ndh*F |
| *M. pruriens* NC_054176.1, *mat*K, 1,512 bp | AT90XM1P014 | *G. soja* PZ683366.1/PV685014.1, *G. max* MZ964143.1 | 100% | 97.35% | 0 | Near-identical to soybean; no *Mucuna among* top hits |
| *M. pruriens* NC_054176.1, *rpo*C1, 2,862 bp | ATABM2CN016 | *C. tetragonoloba* NC_037714.1 and KY241814.1, then *G. soja*/*G. max* (98.01%) | 100% | 98.04% | 0 | Closest to *Cyamopsis*; KY241814.1 is the deposited *C. tetragonoloba* plastome |
| *M. pruriens* NC_054176.1, *ycf*2 main fragment, 6,864 bp | ATABM2CN016 | *G. soja* PV685018.1/PZ683366.1, *G. max* MW357265.1 | 100% | 100.00% | 0 | Identical to soybean *ycf*2; alignment score exceeds the self-hit |
| *M. birdwoodiana* PZ428231.1, *rbc*L (control), 1,428 bp | AT90XM1P014 | Self-hit PZ428231.1; then *M. macrocarpa* (99.93%), *M. sempervirens* (99.72%), *M. manongarivensis* (98.74%), *M. atropurpurea* voucher EU128736.1 (99.71%) | 100% | 99.93% (top congener) | 0 | Control behaves as expected: congeners are the top hits |

**Table S5. Gap/indel burden of the 42,315-bp, 52-gene CDS matrix in each taxon**

| **Taxon** | **Indel columns** | **Note** |
| --- | --- | --- |
| *Mucuna birdwoodiana* PZ428231.1 | 0 | Focal; complete |
| *Mucuna macrocarpa* NC_044116.1 | 0 | Complete |
| *Mucuna sempervirens* NC_063832.1 | 0 | Complete |
| *Mucuna manongarivensis* PZ095201.1 | 4632 | Severely incomplete record (19/76 PCGs absent; ~53%) |
| *Mucuna pruriens* NC_054176.1 | 1929 | Fragmented *ycf*2 and 105-bp *ndh*K annotation |
| *Cajanus cajan* NC_031429.1 | 0 | Complete |
| *Cajanus crassus* NC_057277.1 | 0 | Complete |
| *Cyamopsis tetragonoloba* NC_037714.1 | 0 | Complete |
| *Dolichos falciformis* NC_057447.1 | 0 | Complete |
| *Dunbaria nivea* NC_057448.1 | 18 | Complete; minor indels |
| *Eriosema crinitum* NC_057449.1 | 0 | Complete |
| *Bolusafra bituminosa* NC_057451.1 | 6 | Complete; minor indels |
| *Flemingia macrophylla* NC_065865.1 | 0 | Complete |
| *Flemingia prostrata* NC_065863.1 | 6 | Complete; minor indels |
| *Flemingia stricta* NC_086853.1 | 0 | Complete |
| *Glycine max* NC_007942.1 | 0 | Complete |
| *Glycine soja* NC_022868.1 | 0 | Complete |
| *Glycine tomentella* PQ474252.1 | 9 | Complete; minor indels |
| *Lablab purpureus* MN966634.1 | 6 | Complete; minor indels |
| *Neustanthus phaseoloides* NC_071874.1 | 0 | Complete |
| *Polygala tenuifolia* NC_050829.1 | 762 | Outgroup; elevated divergence (expected) |
| *Rhynchosia minima* PZ168340.1 | 0 | Complete |
| *Sphenostylis angustifolia* PV870736.1 | 3 | Complete; minor indels |
| *Sphenostylis stenocarpa* PP920094.1 | 9 | Complete; minor indels |
| *Vigna unguiculata* KJ468104.1 | 3 | Complete; minor indels |

All ingroup taxa except *M. manongarivensis* and *M. pruriens* contribute ≤18 indel columns; the 19 protein-coding genes absent from *M. manongarivensis* are *acc*D, *atp*F, *cem*A, *clp*P, *mat*K, *ndh*B, *pet*G, *pet*L, *psa*J, *psb*M, *psb*N, *rpl*32, *rpo*C2, *rps*12, *rps*15, *rps*19, *ycf*1, *ycf*3 and *ycf*4.
